# Profiling Serum Cytokines and Anticytokine Antibodies in Psoriasis Patients

**DOI:** 10.1155/2022/2787954

**Published:** 2022-09-08

**Authors:** Dan Hong, Xiuting Liu, Xiaonan Qiu, Siyao Lu, Yanyun Jiang, Guozhen Tan, Zhenrui Shi, Liangchun Wang

**Affiliations:** Department of Dermatology, Sun Yat-sen Memorial Hospital, Sun Yat-sen University, Guangzhou 510120, China

## Abstract

Cytokines like IL-17A have been consistently found to be elevated in psoriatic lesional skin, and therapeutic antibodies to IL-17 have demonstrated efficacy in treating psoriatic skin and joint disease. However, results about the circulating cytokines in psoriasis patients remained controversial. Anticytokine autoantibodies (ACAAs) were detected in various autoimmune diseases but remained largely unknown in psoriasis. We aimed to investigate the serum levels of cytokines and ACAAs in psoriasis patients. The study included 44 biologics-naive psoriasis patients and 40 healthy controls. Serum cytokines and the corresponding autoantibodies were measured by multiplex bead-based technology. The bioactivity of serum IL-17A was determined by IL-8 production in primary keratinocytes. Herein, we found serum levels of IL-12B (median: 6.16 vs. 9.03, *p* = 0.0194) and Th17 cytokines (IL-17A: median: 0.32 vs. 1.05, *p* = 0.0026; IL-22: median: 4.41 vs. 4.41, *p* = 0.0120) were increased in psoriasis patients. More interestingly, bioactive IL-17A was identified in a proportion of patients and positively correlated with disease severity. A few of cytokines were closely associated with each other and formed into a distinct panel in psoriasis. Of 13 anticytokine antibodies, anti-IL-22 was moderately lower (median: 262.8 vs.190.5, *p* = 0.0418), and anti-IL-15 was slightly higher (median: 25.5 vs. 30.5, *p* = 0.0069) in psoriasis than controls. None of ACAAs was related to disease severity. Consequently, the ratios of antibodies to cytokines varied with the pattern of cytokines. In summary, our finding suggested that the levels of circulating bioactive IL-17A were associated with disease activity in psoriasis patients. In contrast, the titers of ACAAs were not significantly altered nor correlated with disease severity. However, the functionality of ACAAs remains to be further demonstrated in vitro in future studies.

## 1. Introduction

Psoriasis is a chronic inflammatory skin disease characterized by epidermal hyperplasia and immune cells infiltration in dermis [1]. Th17 cytokine family (IL-17A, IL-17F, and IL-22) is the major disease driver, whereas Th1 (IL-12, IFN-*γ*), IL-1 cytokine family (IL-1*α*, IL-18), TNF-*α*, IL-6, and neutrophil chemoattractant (IL-8) are recognized as disease contributors [2]. Certain cytokine expression in psoriatic lesions reaches a consensus but not that in blood across previous studies. For instance, some studies reported serum IL-17A was elevated [3–5], whereas others found no differences in psoriasis patients comparing to healthy controls [6, 7]. Accordingly, the associations of serum cytokines with disease severity varied with studies [4, 5, 7].

Anticytokine autoantibodies (ACAAs) were detected in various autoimmune diseases, such as rheumatoid arthritis (RA), systemic sclerosis, multiple sclerosis, and systemic lupus erythematosus (SLE) [8]. For instance, anti-TNF-*α* and anti-IL-17 autoantibodies were detected in RA patients. In addition, anti-IL-17A antibodies were associated with bone destruction and disease activity [9–11]. The generation of ACAAs was due to the loss of B cell tolerance caused by the overexpression of key inflammatory cytokines and chemokines [12]. High-affinity ACAAs may neutralize cytokines and potentially restrain destructive immune response [13]. ACAAs remain largely unknown in psoriasis patients. Only anti-TNF-*α* and anti-IFN-*α* antibodies were reported once [14]. Herein, we examined 13 of psoriasis-related serum cytokines and the corresponding autoantibodies to evaluate their correlations with disease activity, clinical and laboratory parameters in psoriasis patients. The bioactivity of IL-17A was particularly assessed in vitro.

## 2. Materials and Methods

### 2.1. Serum Samples

Psoriasis patients who fulfilled the following criteria were enrolled in the study: psoriasis vulgaris with no psoriatic arthritis; no infectious or immune-related systemic diseases; never received biologics therapy; and off systemic therapies for at least 1 month before enrollment. Disease severity was measured by psoriasis area severity index (PASI) and body surface area (BSA). Approximately 10 mL of blood was obtained from each individual. After centrifugation at 1000×g for 10 min at 4°C, serum was collected under sterile conditions and stored at -80°C until used. Informed consent was obtained from all individual participants included in the study. The study was approved by the research ethics board of Sun Yat-sen Memorial Hospital (2020-KY-017).

### 2.2. Examination of Multiplex Cytokines and ACAAs

Multiplex cytokines and ACAAs were examined using Milliplex Map Kit (Merck Millipore, HCYTA-60K; Merck Millipore, HCYTAAB-17K). The experiments were performed according to the manufacturer's instructions. The Mean Fluorescent Intensity (MFI) data were read out on Luminex MAGPIX® system (Luminex Corp) and converted into concentrations using 5-parameter logistic method for cytokines. The quality control ranges for cytokine detection were generated with overnight assay format using serum matrix provided in the kit. The detectable concentration (DC) for cytokines were shown as follows (pg/mL): TNF-*α* [3.03-96158], IL-12B [0.75-101356], IL-17A [0.64-41995], IL-17F [8.34-1562076], IL-22 [8.82-24291], IL-1*α* [2.39-90500], IL-6 [0.2-33187], IL-8 [0.33-11435], IL-10 [1.26-80974], IL-15 [0.86-109300], IL-18 [0.34-18275], IFN-*γ* [0.89-35417], and G-CSF [1.01-80835]. Values below the minDC were set to 1/2 minDC. Positive and negative control beads were provided in the kit for antibody detection.

### 2.3. Detection of Bioactive IL-17A

Primary keratinocytes (ATCC, PCS-200-010) were seeded at a density of 1 × 10^5^ cells/well in 12-well plates. Serum was diluted with culture medium at a concentration of 10% and incubated with anti-IL-17A antibodies (10 *μ*g/mL, Secukinumab, Novartis Pharmaceuticals Corporation) at 37°C for 1 hour before adding into cell culture. After coculture for 24 hours, the supernatants were collected, and the levels of IL-8 were examined by an enzyme-linked immunosorbent assay kit (Thermo Fisher, 88-8086-22).

### 2.4. Statistical Analysis

The data was analyzed using GraphPad Prism8 (GraphPad Software, San Diego, CA, USA). Two-sided Mann–Whitney *U* test was used to compare two groups. Spearman's correlation test was applied to explore correlations. The bubble chart of correlation matrix across cytokines was performed by R's corrplot package. *p* value < 0.05 was considered statistically significant.

## 3. Results

### 3.1. Patients

There were 44 patients and 40 healthy controls enrolled in this study. The patient cohort consisted of 36 males and 8 females. The mean age was 39.91 ± 11.62 (mean ± SD, range: 21-78) years. The age and gender were matched between patients and controls. The mean duration of illness was 10 ± 6.2 (mean ± SD, range: 1-24) years. The PASI and BSA score were 16.83 ± 7.90 (mean ± SD, range: 2-40.8) and 27.46 ± 17.54 (mean ± SD, range: 1-82.5), respectively.

### 3.2. Profile of Psoriasis-Related Cytokines

**Figure 1:**
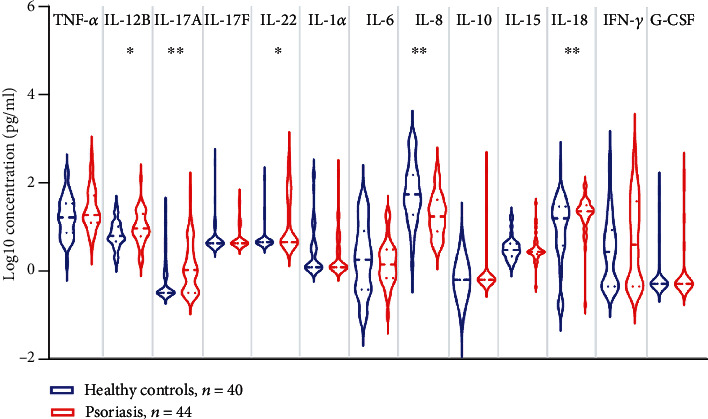
Profile of serum cytokines in healthy controls and psoriasis patients. Serum levels of tumor necrosis factor (TNF-*α*), interleukin- (IL-) 12B, IL-17A, IL-17F, IL-22, IL-1*α*, IL-6, IL-8, IL-10, IL-15, IL-18, interferon gamma (IFN-*γ*), and granulocyte colony-stimulating factor (G-CSF) in healthy controls (HC, *n* = 40) and psoriatic patients (PSO, *n* = 44) are measured by multiplex bead-based technology. Dotted line shows median and quartile. ^∗^*p* < 0.05, ^∗∗^*p* < 0.01.

**Figure 2:**
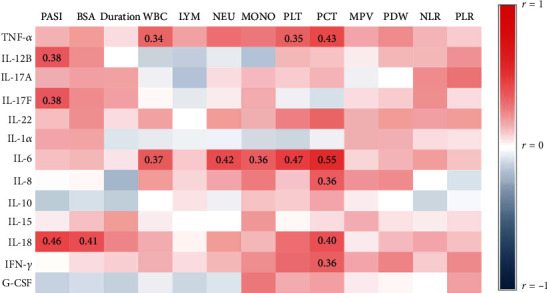
Correlation of serum cytokines with clinical parameters in psoriasis patients. Correlations of serum cytokines with PASI, BSA, duration (*n* = 44), and the absolute number of white blood cell (WBC), lymphocyte (LYM), neutrophil (NEU), monocyte (MONO), platelet (PLT), plateletcrit (PCT), mean platelet volume (MPV), platelet distribution width (PDW), neutrophil-lymphocyte ratio (NLR), and platelet-lymphocyte ratio (PLR) (*n* = 34) are analyzed by Spearman's correlation. Positive correlations are shown as red and negative as blue. The intensity of color is proportional to the correlation coefficients. Statistically significant *r* values (*p* < 0.05) are given in the corresponding rectangle.

**Figure 3:**
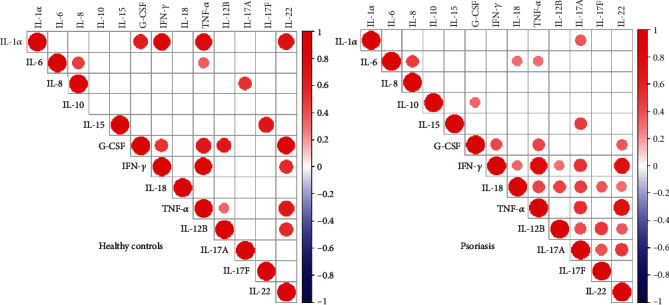
Correlation of serum cytokines in healthy controls (HC) and psoriasis patients (PSO). Correlations of cytokines in (a) HC (*n* = 40) and (b) PSO (*n* = 44) are analyzed by Spearman's correlation. Positive correlations are shown as red and negative as blue. The intensity of color and the size of dots are proportional to the correlation coefficients. Only significant Spearman's correlation coefficients (*p* < 0.05) are shown.

Numerous evidence consistently showed elevated expression of cytokines including Th17-related cytokines, TNF-*α*, IL-1*α*, IL-6, and IFN-*γ* in psoriatic lesion; however, the conflicting results are obtained about their circulating level [6, 7, 15–17]. We first examined the circulating levels of cytokines in our cohort of patients. Serum levels of cytokines were listed in supplementary table 1. Th17-related cytokines including IL-12B (median: 6.16 vs. 9.03, *p* = 0.0194), IL-17A (median: 0.32 vs. 1.05, *p* = 0.0026), and IL-22 (median: 4.41 vs. 4.41, *p* = 0.0120) were moderately higher in psoriasis patients than in healthy controls, whereas sera TNF-*α*, IL-17F, IL-1*α*, IL-6, IL-10, IL-15, IFN-*γ*, and G-CSF were comparable between the two groups. Unexpectedly, serum IL-8, the neutrophil-related chemokine [18], was greatly reduced in psoriasis patients (Figure 1). We then analyzed the correlation of cytokines with clinical and laboratory parameters (Figure 2) and observed that serum IL-18 was positively correlated with disease severity represented by PASI (*r* = 0.46, *p* = 0.0018) and BSA (*r* = 0.41, *p* = 0.0065). IL-12B (*r* = 0.38, *p* = 0.0111) and IL-17F (*r* = 0.38, *p* = 0.0106) were positively related to PASI, but not to BSA. Both TNF-*α* and IL-6 were positively associated with the total counts of white blood cell (WBC) (TNF-*α*: *r* = 0.34, *p* = 0.0495; IL-6: *r* = 0.37, *p* = 0.0328), platelet (PLT) (TNF-*α*: *r* = 0.35, *p* = 0.0455; IL-6: *r* = 0.47, *p* = 0.0047), and plateletcrit (PCT) (TNF-*α*: *r* = 0.43, *p* = 0.0134; IL-6: *r* = 0.55, *p* = 0.0011). Additionally, IL-6 was correlated with neutrophils (*r* = 0.42, *p* = 0.0134) and monocytes (*r* = 0.36, *p* = 0.0376) while IL-8 (*r* = 0.36, *p* = 0.0413), IL-18 (*r* = 0.40, *p* = 0.0243), and IFN-*γ* (*r* = 0.36, *p* = 0.0459) were positively correlated with PCT. Surprisingly, none of these cytokines were related to the counts of lymphocytes. We next analyzed the correlations across cytokines, because cytokines such as IL-17A and TNF-*α* were well known to work synergistically in human keratinocytes to promote inflammation in psoriasis [19]. Of considerable interest, the correlation matrix revealed a distinct cytokine panel where cytokines (IFN-*γ*, IL-18, TNF-*α*, IL-12B, IL-17A, IL-17F, and IL-22) were positively correlated with each other in psoriasis patients but not in healthy controls (Figure 3).

In particular, serum levels of IL-17A were strongly correlated with IL-1*α*, IL-15, IFN-*γ*, IL-18, TNF-*α*, IL-12B, IL-17F, and IL-22 in psoriasis but not in healthy controls. Overall, these findings suggest that a panel of cytokines dominated by Th17 family is elevated and formed into a network in a close relationship in serum of psoriasis patients.

### 3.3. Correlation of Bioactive IL-17A with Psoriasis Disease Activity

**Figure 4:**
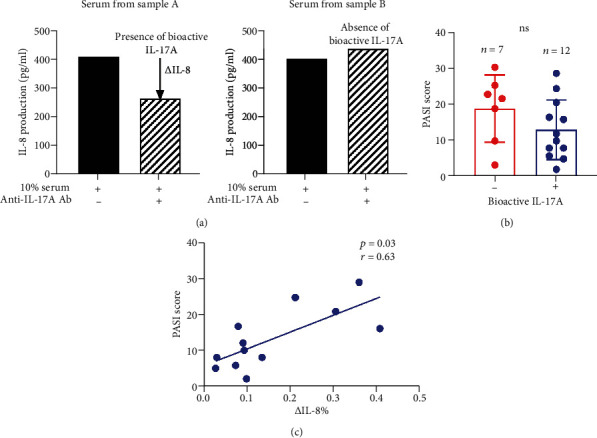
Bioactivities of serum IL-17A were correlated with psoriasis severity. (a) Primary keratinocytes (PKC) were cocultured with serum diluted in 10% in the presence and absence of anti-IL-17A antibodies. IL-8 in medium was measured by ELISA 24 hours later. (b) The comparison of the PASI score between patients with or without bioactive IL-17A in serum. (c) Correlation analysis between PASI score and the percentage of the decreased levels of IL-8 (*Δ*IL-8) relative to those cultured with serum. Data are presented as mean ± SD and compared by the Mann–Whitney *U* test in (b) and Spearman's correlation test in (c). ns: not significant.

IL-17A overexpression is consistently detected in psoriatic lesions and therapeutic antibodies against IL-17A achieve great success in treating psoriasis [20], suggesting a pivotal role of IL-17A in psoriasis pathogenesis. We showed serum IL-17A was increased but not correlated with disease activity (Figures 1 and 2), which raised an interesting question of whether the proportion of bioactive IL-17A varied with each patient while total IL-17A was maintained at a certain level. IL-8 was suggested to be a sensitive marker to measure the strength of IL-17A stimulation [21]. To test our hypothesis, we measured the bioactivity of serum IL-17A as previously reported [22]. Herein, the level of IL-8 produced by primary keratinocytes in response to serum sample stimulation was taken as the bioactivity of serum IL-17A. To determine the contribution of IL-17A, serum samples were preincubated with and without anti-IL-17A antibodies, diluted into the medium before cell culture. IL-8 in the supernatant was measured by ELISA 24 hours later. As shown in Figure 4(a), IL-8 production stimulated by serum sample A was greatly reduced by pretreatment with anti-IL-17A antibodies, indicating the presence of bioactive IL-17A in sample A. But no reduction was observed in serum sample B pretreated with anti-IL-17A antibodies, indicating that IL-8 production was attributed to factors other than serum IL-17A. Based on the test, patients were divided into two groups with and without bioactive IL-17A. Although, the PASI score was comparable between the two groups (Figure 4(b)), the degree of IL-8 reduction was positively related to the PASI score (*p* = 0.03, *r* = 0.63) (Figure 4(c)), suggesting bioactive IL-17A is positively correlated with psoriasis disease activity.

### 3.4. Profile of Serum ACAAs in Psoriasis Patients

**Figure 5:**
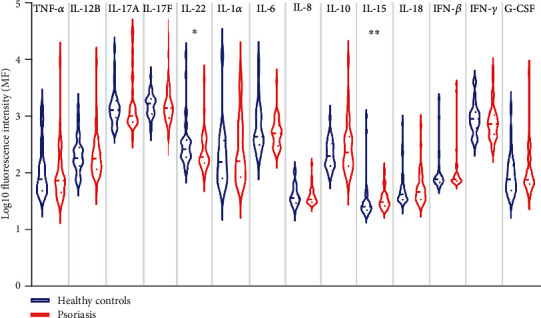
Profile of serum autoantibodies against cytokines (ACAAs) in healthy controls and psoriasis patients. Mean fluorescence intensity (MFI) of autoantibodies against TNF-*α*, IL-12B, IL-17A, IL-17F, IL-22, IL-1*α*, IL-6, IL-8, IL-10, IL-15, IL-18, IFN-*β*, IFN-*γ*, and G-CSF in HC (*n* = 40) and PSO (*n* = 44) are measured by multiplex bead-based technology. Dotted line shows median and quartile. ^∗^*p* < 0.05, ^∗∗^*p* < 0.01.

**Figure 6:**
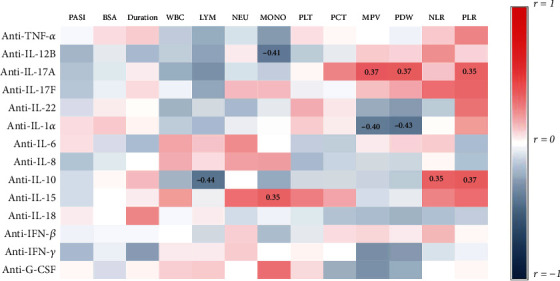
Correlation of serum autoantibodies against cytokines (ACAAs) with clinical parameters in psoriasis patients. Correlations between MFI of auto Abs and PASI, BSA, duration (*n* = 44), and the absolute number of white blood cell (WBC), lymphocyte (LYM), neutrophil (NEU), monocyte (MONO), platelet (PLT), plateletcrit (PCT), mean platelet volume (MPV), platelet distribution width (PDW), neutrophil-lymphocyte ratio (NLR), and platelet-lymphocyte ratio (PLR) (*n* = 34) are analyzed by Spearman's correlation. Positive correlations are shown as red, and negative ones are shown as blue. The intensity of color is proportional to the correlation coefficients. Statistically significant *r* values (*p* < 0.05) are given in the corresponding rectangle.

Emerging evidence highlights a beneficial and protective effect of autoantibodies against pathogenic cytokines especially in inflammatory disease [12]. We assessed serum antiantibodies against the aforementioned cytokines in the same two cohorts (Figure 5). Serum levels of autoantibodies varied broadly across patients and controls (listed in supplementary table 2). For instance, the mean fluorescence intensity (MFI) of anti-TNF-*α* autoantibodies ranged from 28 to 9536. Anti-IL-22 (median: 262.8 vs.190.5, *p* = 0.0418) antibody was decreased whereas anti-IL-15 (median: 25.5 vs. 30.5, *p* = 0.0069) was increased in psoriasis patients compared to that in healthy controls. Autoantibodies against other cytokines were found no significant differences between the two groups. Anti-IL-17A was positively correlated with MPV, PDW, and PLR. Besides, anti-IL-10 was negatively correlated with LYM, while positively correlated with NLR and PLR (Figure 6). Since ACAAs had no associations with psoriasis disease activity, we hypothesized that a panel of autoantibodies, rather than a single antibody, might be required to restrain the proinflammatory process of psoriasis, which is in correspondence with the synergetic working pattern of cytokines. To test the hypothesis, we defined autoantibodies above the median levels of the entire population as positive and below that as negative. Surprisingly, patients either with negative anti-IL12B and anti-IL-22 (median, 21.8 vs. 16.4, *p* = 0.012) or with negative anti-12B and anti-IL-17A (median, 22.0 vs. 16.5, *p* = 0.032) presented higher PASI score than those with positive antibodies (data not shown). Together, these findings showed that the major profile of ACAAs is at a normal range except anti-IL-22 and anti-IL-15 antibodies, and the titers of single ACAA hardly correlated with psoriasis severity.

### 3.5. Balance of ACAAs and Cytokines in Psoriasis Patients

Finally, we evaluated the balance of autoantibodies and cytokines by comparing the ratio of autoantibodies to cytokines. Remarkably, the ratios of autoantibodies to cytokines of IL-22, IL-17A, and IFN-*γ* were significantly decreased, whereas the ratio of anti-IL-8 antibody to IL-8 was elevated in patients with psoriasis (supplementary materials: Figure S1). The correlation of autoantibody/cytokine ratio with clinical severity shared the similar pattern with what was observed in cytokines (supplementary materials: Figure S2). Exclusive relationship of IL-17F pair with PLR was observed, which was not found in cytokine or autoantibody alone. These findings suggested that the imbalance of autoantibodies to cytokines mainly attributes to cytokines variation.

## 4. Discussion

ACAAs occur across various infectious diseases, cancer, and autoimmune diseases and can be beneficial or detrimental in various contexts. As for cases with immunodeficiency, ACAAs may be causative or closely associated with susceptibility to mucocutaneous candidiasis or *Talaromyces marneffei* by neutralizing cytokines [23, 24]. SLE patients with anti-IFN-*α* antibodies presented less active IFN-pathway and low disease activity [25]. Although anticytokine antibodies achieved great success in treating psoriasis, the profile of serum ACAAs was largely unknown. We herein showed that of 13 ACAAs examined in this study, only anti-IL-22 and anti-IL-15 in psoriasis differed from those in controls. Elevated anti-TNF-*α* antibody was once reported in psoriasis [14]. In disagreement, we showed anti-TNF-*α* in psoriasis did not differ from controls, which could attribute to cohorts of patients and techniques of antibody examination. We used bead multiplex assays to detect both cytokines and ACAAs simultaneously. Theoretically, it limited variations that potentially resulted from each run [26].

IL-22 belongs to IL-10 family released by Th17, Th22, NKT, and *γδ* T cells. IL-22 mediates IL-23-induced acanthosis and dermal inflammation by activating Stat3 pathway in vivo [27]. Neutralization of IL-22 prevents the development of psoriasis-like inflammation in mice [28]. IL-22 expression was upregulated both in serum and psoriatic lesions [29]. We posited the low anti-IL-22 antibodies might contribute to the development of psoriasis due to failure of reducing excessive production of this cytokine. Although no associations were observed between disease severity with any of the ACAAs alone including anti-IL-22 Abs, we found that patients that lack a panel of auto Abs to both IL-12B/IL-22 or IL-12B/IL-17A tended to have higher PASI score, possibly suggesting a combinative effects of auto Abs to suppress the inflammation. However, further studies are warranted to prove neutralizing activities of auto Abs using functional in vitro assays.

IL-17 mRNA and protein levels have also been demonstrated to be measurably higher in lesional psoriatic tissue than in nonlesional tissue thus correlating IL-17 to psoriatic disease [30]. Within the skin, IL-17A acts on cellular targets, including keratinocytes, neutrophils, endothelial cells, and fibroblasts and stimulates the production of various antimicrobial peptides, chemokines, and proinflammatory and proliferative cytokines, which, in turn, promote tissue inflammation. IL17A-targeting antibodies show an impressive clinical efficacy in patients with psoriasis. Studies have reported an improvement of at least 75% as measured by the PASI score in >80% of patients treated with anti-IL-17A therapy [31, 32]. Unexpectedly, controversial results have been reported about the serum levels of IL-17A and its associations with disease activity. Despite the well-evidenced therapeutic efficacy of biologic agents targeting IL-17A, several studies found comparable levels of IL-17A between psoriasis and healthy donors [6, 7]. One possibility behind such conflicting results is that the bioactivity of IL-17A differs within the group of psoriasis patients. Our results demonstrated that the bioactivity of serum IL-17A varies among different individual patients. Particularly, we observed a positive correlation between IL-17A bioactivity and skin severity whereas such a relationship was not observed using the total amount of IL-17A. In the future, it will be tempting to further investigate the association between bioactive serum IL-17A with the response or relapse to anti-17A biologics. Also, since emerging evidences highlighted the role of serum IL-17 in vascular damage [33], bioactive IL-17A should be taken into count.

IL-17 is well known for its synergistic interactions with other cytokines, such as TNF-*α*. For example, IL-17 upregulated TNF receptor II (TNFR-II) expression in synoviocytes and acted together with TNF to contribute rheumatoid arthritis [34]. Other mechanisms include increased mRNA stability, notably for IL-6 and IL-8 [35]. Indeed, we found more positive correlations between cytokines, especially IL-17A with other cytokines such as TNF-*α* in psoriasis than those in healthy donors, supporting the notion for treatment with dual inhibition of IL-17A and TNF-*α* (or other cytokines) in psoriasis patients resistant to single biologics.

To be noted, previous studies have shown that many cytokines including IL-17A are more enriched in the psoriatic lesion than in the circulation, implying a more critical role of local cytokines than circulating ones in the development of skin damage [3, 36]. Despite statistical significance, the fold changes of many circulating cytokines examined in our study is relatively small. Together with their weak correlation with disease severity, these changes could be biologically irrelevant for cutaneous damage. However, it would be of interest to further study the associations between circulating cytokines and systemic inflammation marker like erythrocyte sedimentation rate (ESR), C-reactive protein (CRP), and amyloid A (SAA).

Limitations of the study were the small sample size and the potential immune complex of cytokines and anticytokine antibodies captured by bead multiplex assays. Also, the functionality and clinical significance of anticytokine autoantibodies requires further investigation. In sum, ACAAs were not significantly altered nor correlated with disease activity in psoriasis patients. Interestingly, we revealed bioactive form but not the total amount of IL-17A in the circulation was correlated with disease activity. These results need to be extended to larger populations of psoriasis and other IL-17-driven diseases, specifically using samples for which the response to an IL-17 inhibitor is known.

## Data Availability

No datasets were generated during the current study, but some or all data are available from the corresponding authors by request.

## Abbreviations

Auto Abs: Autoantibodies
ACAAs: Anticytokine autoantibodies
BSA: Body surface area
G-CSF: Granulocyte colony-stimulating factor
IL: Interleukin
LYM: Lymphocyte
MFI: Mean fluorescence intensity
MONO: Monocyte
MPV: Mean platelet volume
NEU: Neutrophil
NLR: Neutrophil-lymphocyte ratio
PKC: Primary keratinocytes
PASI: Psoriasis area and severity index
PLT: Platelet
PCT: Plateletcrit
PDW: Platelet distribution width
PLR: Platelet-lymphocyte ratio
SLE: Systemic lupus erythematosus
Th: T helper
TNF-*α*: Tumor necrosis factor
WBC: White blood cell.

## Supplementary Materials

Supplementary Materials Table S1: serum levels of cytokines in healthy controls and psoriatic patients. Table S2: serum levels (MFI) of autoantibodies in healthy controls and psoriatic patients. Figure S1: profile of autoantibodies/cytokines in healthy controls and psoriatic patients. Figure S2: correlation matrix between MFI ratio of autoantibodies/cytokines and clinical parameters.
