# Supplementary material for: Profiling Serum Cytokines and Anticytokine Antibodies in Psoriasis Patients
Source: J Immunol Res. 2022 Sep 8;2022:2787954. doi: 10.1155/2022/2787954 (PMC9477620; doi:10.1155/2022/2787954)
Supplement: Supplementary Materials — Table S1: serum levels of cytokines in healthy controls and psoriatic patients. Table S2: serum levels (MFI) of autoantibodies in healthy controls and psoriatic patients. Figure S1: profile of autoantibodies/cytokines in healthy controls and psoriatic patients. Figure S2: correlation matrix between MFI ratio of autoantibodies/cytokines and clinical parameters. [file 2787954.f1.pdf]

Table S1 Serum levels of cytokines in healthy controls and psoriatic patients

	**HC, n=40**		**PSO, n=44**		
pg /ml	mean ± SD	median[min-max]	mean ± SD	median[min-max]	*p-value
TNF-α	28.02±35.42	16.14[1.515-169.36]	48.35±77.99	18.12[3.46-438.38]	0.1157
**IL-12B**	8.05±6.01	6.16[1.6-29.72]	14.38±18.73	9.03[1.6-121.78]	**0.0194**
**IL-17A**	1.66±4.37	0.32[0.32-25.37]	3.45±8.48	1.05[0.32-54.22]	**0.0026**
IL-17F	12.78±43.98	4.17[4.17-281.39]	7.45±8.37	4.17[4.17-40.45]	0.8081
**IL-22**	8.27±19.64	4.41[4.41-127.33]	39.12±74.73	4.41[4.41-396.45]	**0.0120**
IL-1α	12.51±34.11	1.195[1.195-156.54]	6.86±23.85	1.195[1.195-158.68]	0.2055
IL-6	7.42±12	1.77[0.1-49.91]	2.85±4.36	1.385[0.1-19.31]	0.2837
**IL-8**	175.72±275.58	53.655[1.29-1053]	33.78±45.58	16.805[2.82-233.21]	**0.0093**
IL-10	0.63±0	0.63[0.63-0.63]	5.67±29.85	0.63[0.63-197.64]	0.5112
IL-15	3.83±3.3	2.925[1.15-17.51]	4.14±5.28	2.66[0.43-33.87]	0.7746
**IL-18**	22.52±34.79	15.33[0.17-212.1]	24.95±17.18	22.075[0.17-84.78]	**0.0069**
IFN-γ	18.59±54.15	2.64[0.445-293.6]	39.77±94.27	3.955[0.445-522.48]	0.2055
G-CSF	2.95±11.13	0.505[0.505-66.41]	6.33±26.37	0.505[0.505-157]	0.9604

*Data are compared by Mann-Whitney U test.

Table S2 Serum levels(MFI) of autoantibodies in healthy controls and psoriatic patients

	HC, n=40		PSO, n=44		
	mean ± SD	median[min-max]	mean ± SD	median[min-max]	*p-value
Anti-TNF-α	222.76±344.86	78[36-1423]	332.47±1425.3	73[28-9536]	0.3955
Anti-IL-12B	282.46±290.21	181.5[65-1550]	451.61±1363.88	179[51-9155.5]	0.6130
Anti-IL-17A	2010.25±2655.08	1315.5[537.5-15932]	3631.43±7462.07	1039.5[430-33809]	0.3208
Anti-IL-17F	1761.13±841.1	1714.5[567-5111.5]	2806.69±4349.58	1421[424-25377]	0.7409
**Anti-IL-22**	759.78±2071.71	262.75[107.5-12433]	360.48±667.65	190.5[86-4484.5]	**0.0418**
Anti-IL-1α	800.23±2615.71	156[42-12656]	727.61±1337	161.75[51-6511]	0.3341
Anti-IL-6	1063.45±1767.91	445.25[163.5-9790.5]	623.78±606.81	509.25[206-4158]	0.6225
Anti-IL-8	43.64±21.91	36[20-112.5]	40.68±19.65	34.25[26.5-143.5]	0.7196
Anti-IL-10	248.41±166.9	198[87-966]	594.20±1497.53	230.25[61-9882]	0.3386
**Anti-IL-15**	66.41±178.52	25.5[17-1038]	37.27±19.19	30.5[21.5-113]	**0.0069**
Anti-IL-18	77.71±124.51	42[27-754.5]	74.19±107.39	46.25[26-700]	0.4000
Anti-IFN-β	157.1±347.03	77[53-2105]	230.98±677.51	77[62-3725]	0.4576
Anti-IFN-γ	1156.93±961.94	901[308.5-4252]	1060.14±1159.38	735.75[258-6589]	0.2297
Anti-G-CSF	135.69±222.12	77[32-1381]	244.05±862.21	76[39-5805]	0.2558

*Data are compared by Mann-Whitney U test.

Figure S1. profile of autoantibodies/cytokines in healthy controls and psoriatic patients

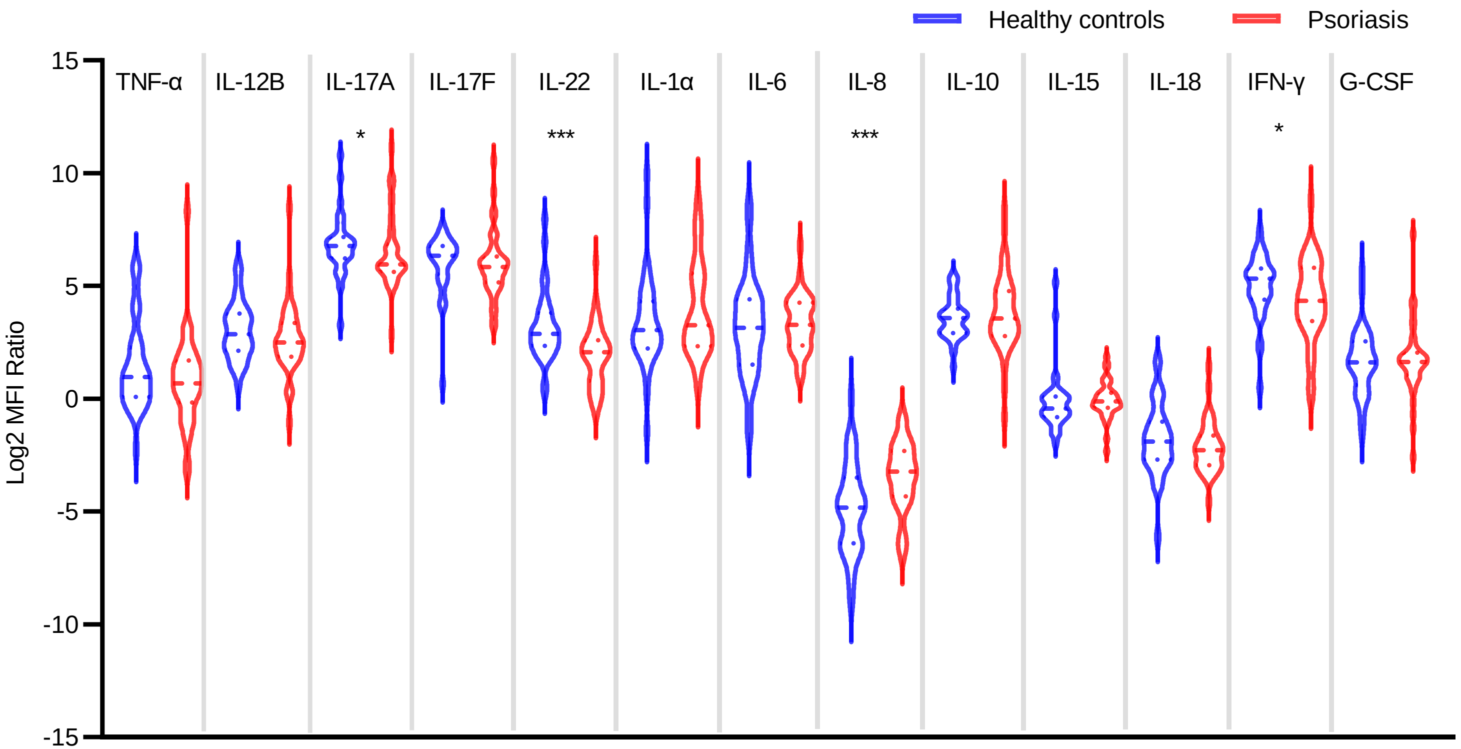

MFI ratio of auto Abs/cytokines against TNF-α, IL-12B, IL-17A, IL-17F, IL-22, IL-1α, IL-6, IL-8, IL-10, IL-15, IL-18, IFN-β, IFN-γ and G-CSF in HC (n=40) and PSO (n=44) are shown. Dotted line shows median and quartile. *p < 0.05, **p < 0.01 , ***p < 0.001.

Figure S2. Correlation matrix between MFI ratio of autoantibodies/cytokines and clinical characteristics

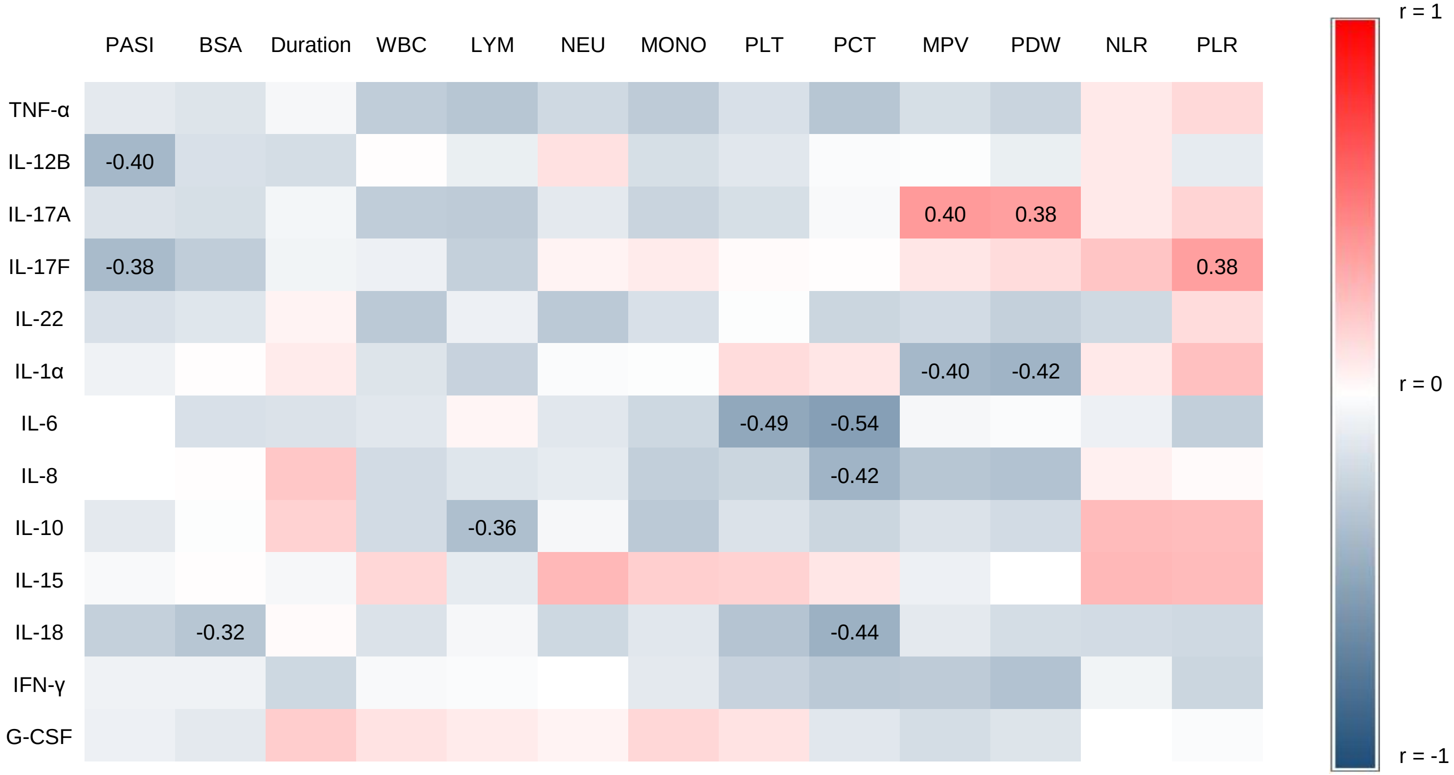

Correlations between MFI ratio and PASI, BSA, duration (n=44), and the absolute number of white blood cell (WBC), lymphocyte (LYM), neutrophil (NEU), monocyte (MONO), platelet (PLT), plateletcrit (PCT), mean platelet volume (MPV), platelet distribution width (PDW), neutrophil-lymphocyte ratio (NLR), platelet-lymphocyte ratio (PLR)(n=34), are analyzed by Spearman correlation. Positive correlations are shown as red and negative ones are shown as blue. The intensity of color is proportional to the correlation coefficients. Only significant Spearman's correlation coefficients ($p < 0.05$) are shown.
